# Supplementary material for: COFFEE: consensus single cell-type specific inference for gene regulatory networks
Source: Brief Bioinform. 2024 Sep 23;25(6):bbae457. doi: 10.1093/bib/bbae457 (PMC11418232; doi:10.1093/bib/bbae457)
Supplement: supplementary_bbae457 [file supplementary_bbae457.pdf]

COFFEE: Consensus Single Cell-Type Specific Inference for Gene Regulatory Networks

Musaddiq K Lodi
Integrative Life Sciences
Virginia Commonwealth University
Richmond, VA 23284, United States
lodimk2@vcu.edu

Anna Chernikov
Center for Biological Data Science
Virginia Commonwealth University
Richmond, VA 23284, United States
lodimk4@vcu.edu

Preetam Ghosh
Department of Computer Science
Virginia Commonwealth University
Richmond, VA 23284, United States
pghosh@vcu.edu

1 Supplementary Materials

**Algorithm**	**hESC**		**hHep**		**mDC**		**mESC**	
	**AUPRC**	**AUROC**	**AUPRC**	**AUROC**	**AUPRC**	**AUROC**	**AUPRC**	**AUROC**
COFFEE	0.00510837	0.67872565	0.00245893	0.62501051	0.0001347	0.60260742	0.003422669	0.617723301
GENIE3	0.00150732	0.52129511	0.00199529	0.65956383	0.00013847	0.63416206	0.002983426	0.619933633
GRNBOOST2	0.00183745	0.61518074	0.00223538	0.64827075	0.00022685	0.69357867	0.003499956	0.620294026
GRNVBEM	NA	0.NA	0.03296306	0.52661940	0.500042	0.5	0.000796019	0.498641805
LEAP	0.00158677	0.59095306	0.00373730	0.73537248	0.0000487	0.29709379	0.002282376	0.569046340
PIDC	0.00255958	0.59552885	0.00159831	0.56889242	0.00014493	0.68258511	0.003371424	0.646667434
PPCOR	0.18547707	0.70213309	NA	NA	NA	NA	NA	NA
SCODE	0.00093786	0.50974079	0.00096875	0.53613360	0.0000704	0.45922102	0.003672028	0.651056418
SCRIBE	0.00124305	0.47078903	0.00082174	0.50238626	0.000093	0.55326797	0.001432764	0.471428960
SINCERITIES	0.00070526	0.37034680	NA	NA	NA	NA	NA	NA

Table S1: Evaluation of AUPRC and AUROC for Experimental Datasets: Non-Specific Ground Truth

Algorithm	hESC		hHep		mDC		mESC	
	**AUPRC**	**AUROC**	**AUPRC**	**AUROC**	**AUPRC**	**AUROC**	**AUPRC**	**AUROC**
COFFEE	0.010527308	0.595763901	0.003377432	0.523924452	1.62E-05	0.311954605	0.024792069	0.521726507
GENIE3	0.006750371	0.674351365	0.003318655	0.604359868	0.00011376	0.352423884	0.020848298	0.552828866
GRNBOOST2	0.006250664	0.598308244	0.003402258	0.53171015	0.000121081	0.42628483	0.021006801	0.537071541
GRNVBEM	NA	NA	0.001304026	0.499560846	0.500084002	0.5	0.00876821	0.498619763
LEAP	0.007363484	0.667789738	0.00232282	0.469145824	9.56E-05	0.194150704	0.020713229	0.546105078
PIDC	0.004454772	0.593154267	0.003941492	0.549891766	0.000161155	0.520694059	0.021208326	0.538306354
PPCOR	0.075420994	0.545477038	NA	NA	NA	NA	NA	NA
SCODE	0.004626411	0.611895913	0.002218586	0.462934832	0.000118643	0.355840417	0.031494739	0.603804518
SCRIBE	0.004453981	0.594193173	0.003382654	0.573949982	0.000140759	0.472796136	0.01849361	0.499193401
SINCERITIES	0.002778774	0.4417388	NA	NA	NA	NA	NA	NA

Table S2: Evaluation of AUPRC and AUROC for Experimental Datasets: Specific Ground Truth

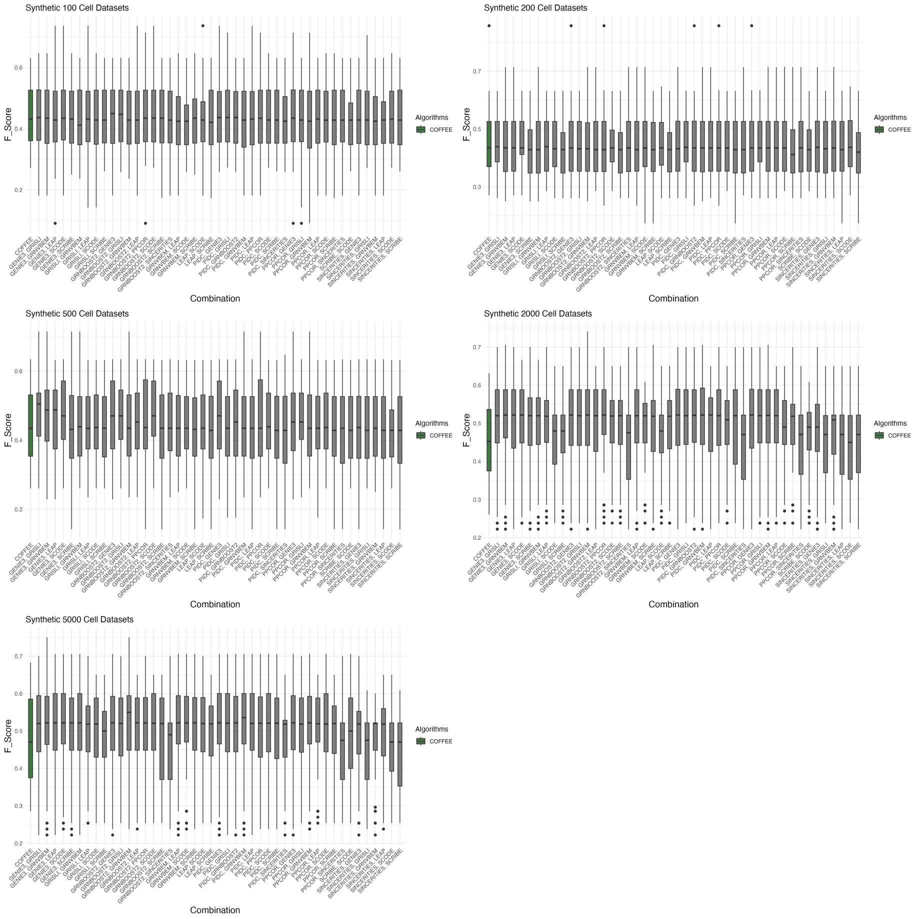

Figure S1: F-Score Performance for Different Combinations of Algorithms in COFFEE vs COFFEE with all 10 Algorithms, in green. The labels correspond to algorithms removed from the original 10 in the COFFEE framework

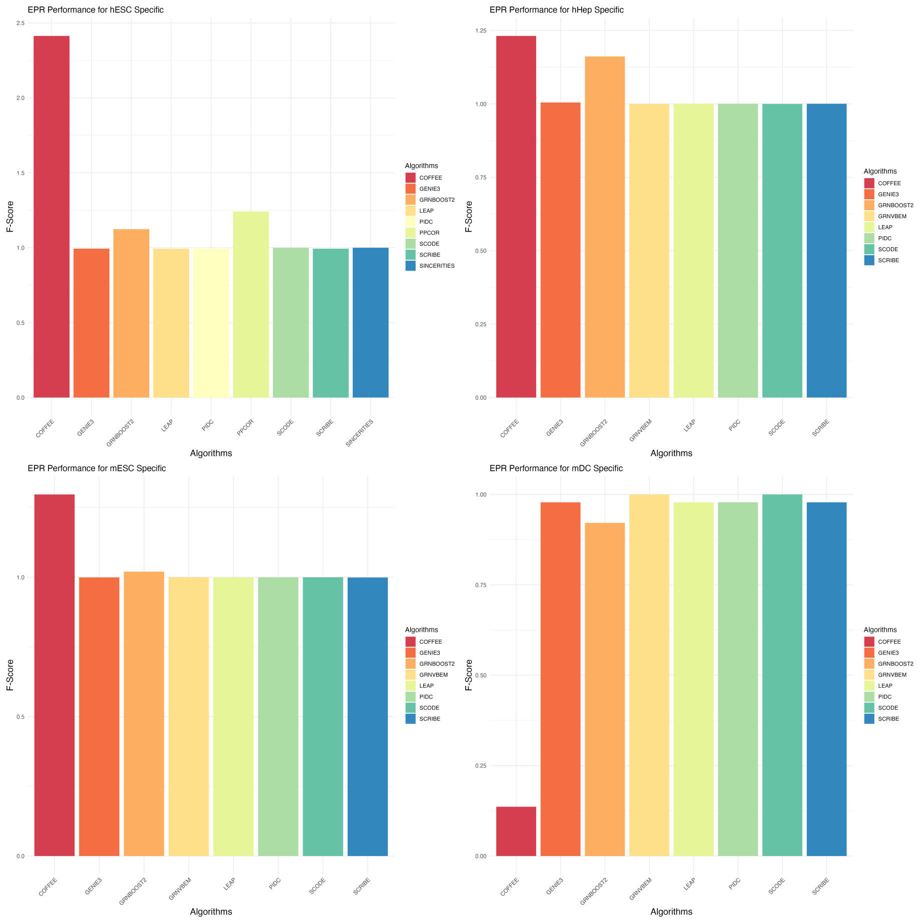

Figure S2: EPR Performance Across Experimental Datasets, Cell-Type Specific Ground Truth

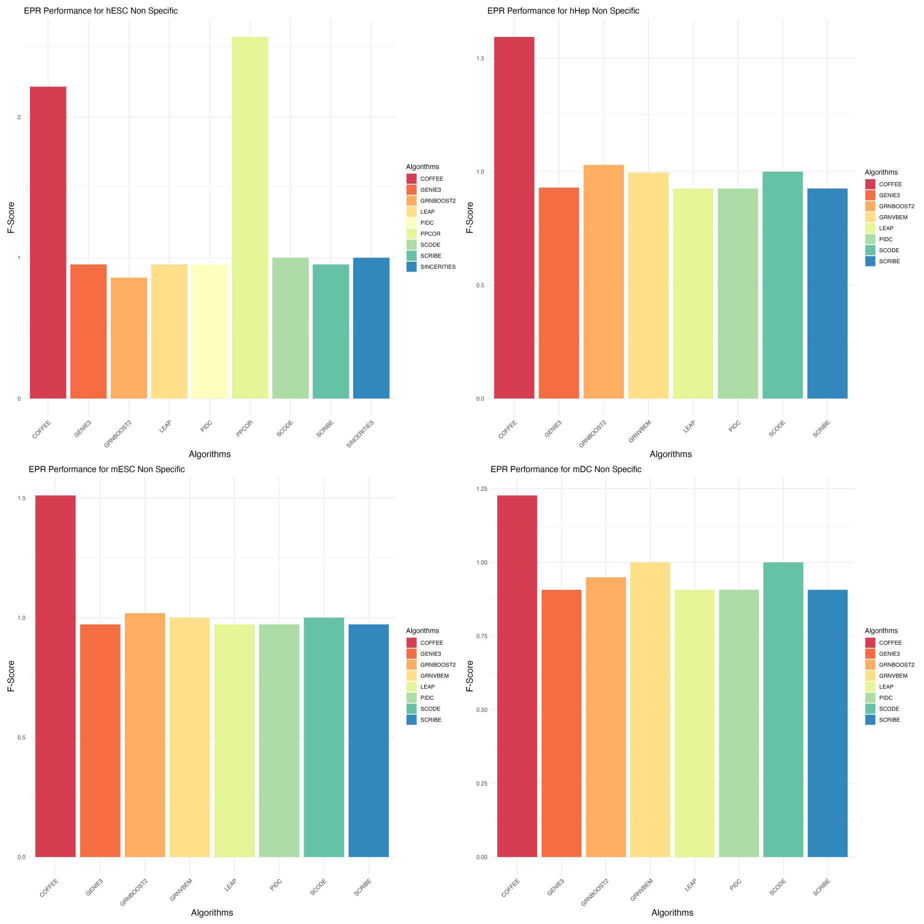

Figure S3: EPR Performance Across Experimental Datasets, Non-Specific Ground Truth

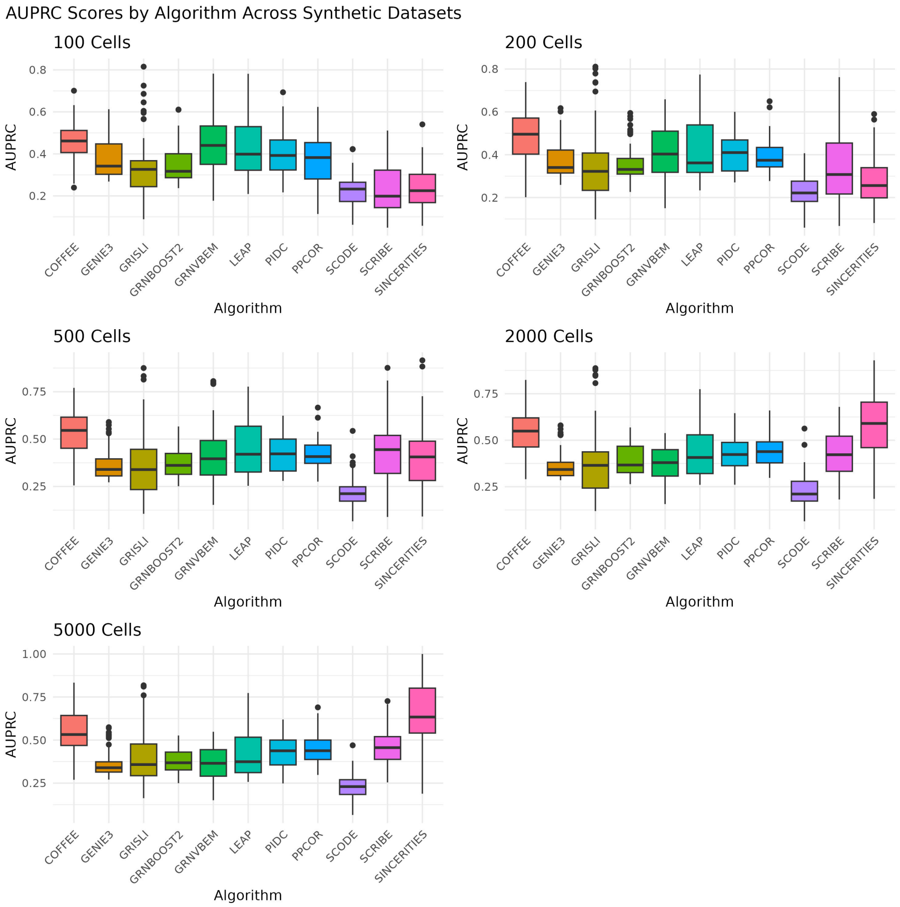

Figure S4: AUPRC Performance Across Synthetic Datasets

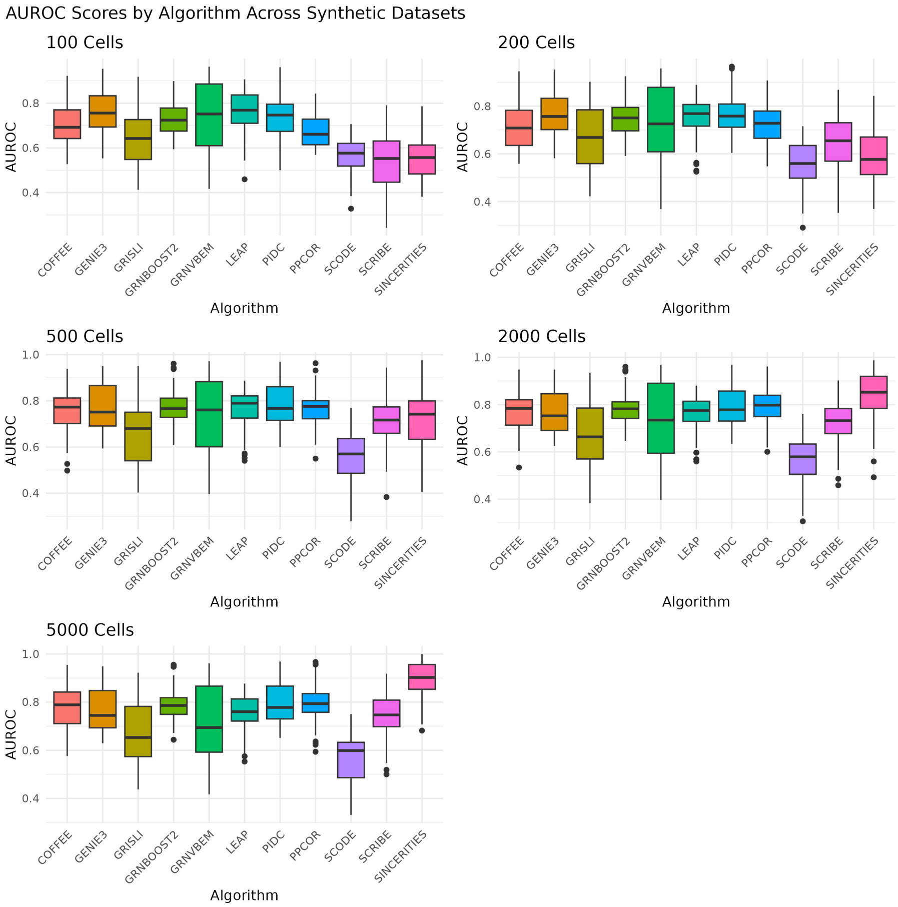

Figure S5: AUROC Performance Across Synthetic Datasets

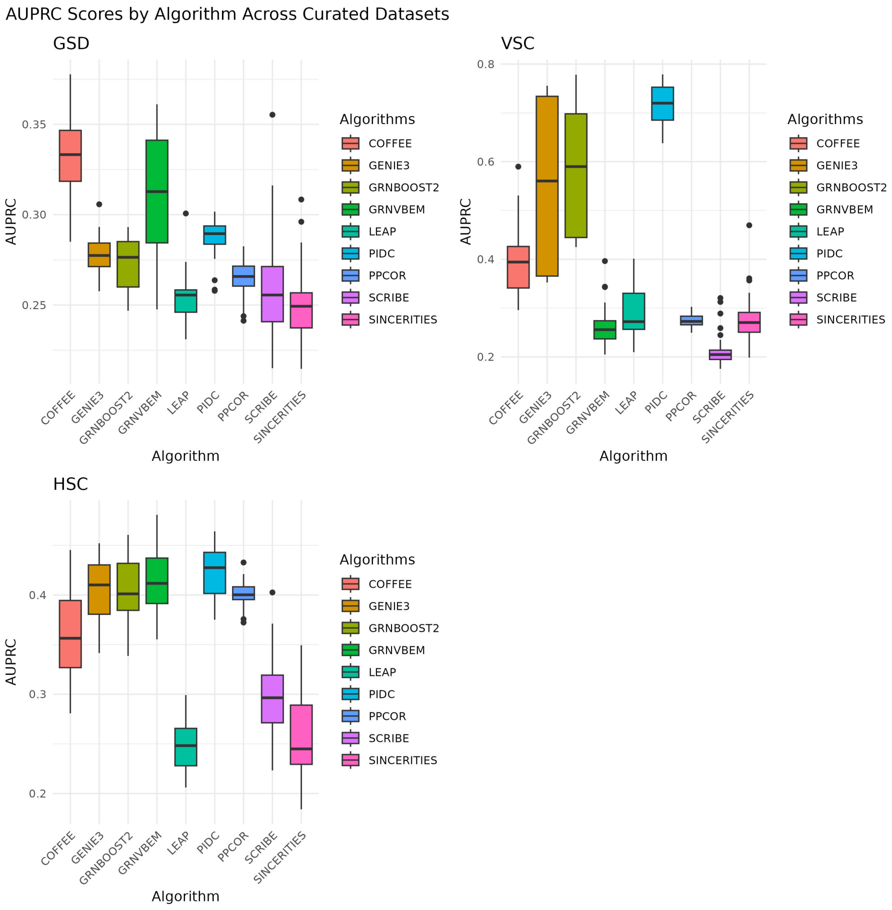

Figure S6: AUPRC Performance Across Curated Datasets

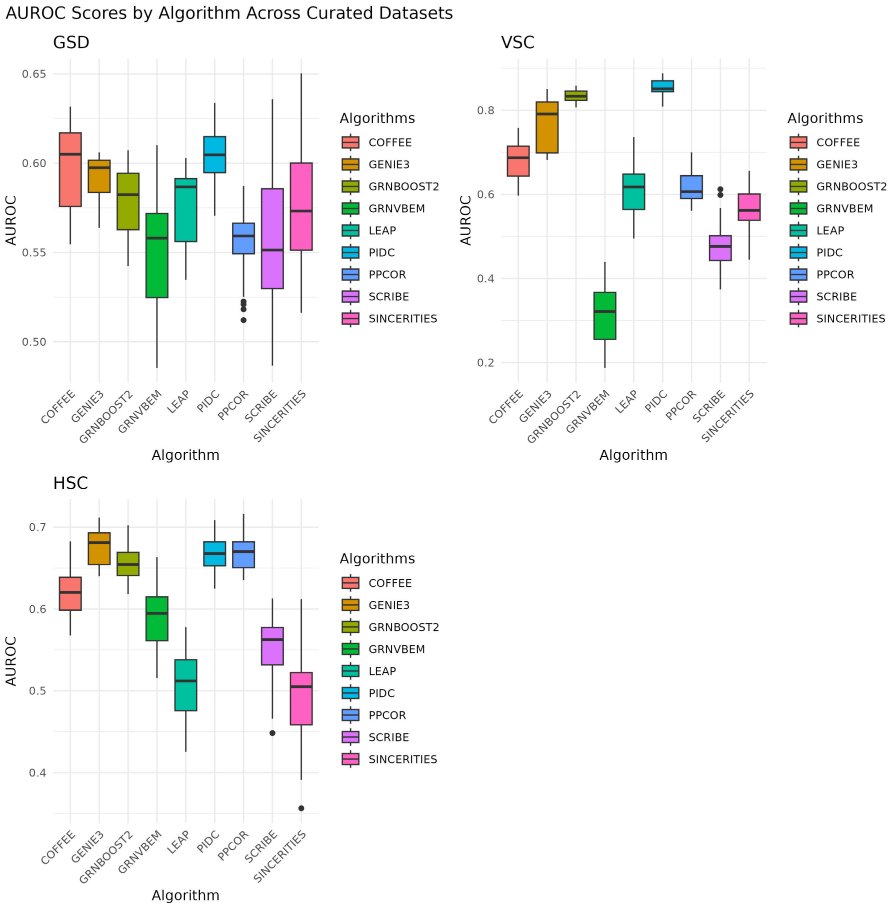

Figure S7: AUROC Performance Across Curated Datasets
